# Vitamin A Nutritional Status of Urban Lactating Chinese Women and Its Associated Factors

**DOI:** 10.3390/nu14153184

**Published:** 2022-08-03

**Authors:** Chenlu Yang, Ai Zhao, Zhongxia Ren, Jian Zhang, Peiyu Wang, Yumei Zhang

**Affiliations:** 1 Department of Nutrition and Food Hygiene, School of Public Health, Peking University, Beijing 100191, China; yangchenluwork@126.com (C.Y.); renzhongxia@bjmu.edu.cn (Z.R.); zhangjian92@pku.edu.cn (J.Z.); 2 Children’s Health Care Center, Beijing Children’s Hospital of Capital Medical University, National Center for Children’s Health, Beijing 100045, China; 3 Vanke School of Public Health, Tsinghua University, Beijing 100091, China; aizhao18@tsinghua.edu.cn; 4 Department of Social Medicine and Health Education, School of Public Health, Peking University, Beijing 100191, China; wpeiyu@bjmu.edu.cn; 5 Beijing Key Laboratory of Toxicological Research and Risk Assessment for Food Safety, School of Public Health, Peking University, Beijing 100191, China

**Keywords:** vitamin A, lactating women, dietary vitamin A, serum vitamin A, breast milk vitamin A, overweight or obese, parity

## Abstract

We aimed to investigate dietary vitamin A (DVA) intake, serum vitamin A (SVA) concentrations, and breast milk vitamin A (BMVA) concentrations of urban lactating Chinese women and explore the associated factors. We recruited 326 lactating women from ten cities in China and collected their dietary information, blood samples, and breast milk samples. SVA and BMVA were assessed with high-performance liquid chromatography. Mann–Whitney U tests, Kruskal–Wallis H tests, Chi-square tests, Spearman correlation tests, and multiple regression analyses were conducted. The median (25th, 75th) of DVA, SVA and BMVA were 372.36 (209.12, 619.78) μg RAE/day, 1.99 (1.71, 2.35) μmol/L, and 1.67 (1.13, 2.15) μmol/L, respectively. Only 3.1% of lactating women met the recommended nutrient intake of Vitamin A (VA), and 20.6% had a BMVA level below 1.05 μmol/L. Compared to underweight or normal weight women, overweight or obese lactating women had lower DVA and BMVA but higher SVA (*p* = 0.022; *p* = 0.030; *p* = 0.003). Multiparous women had a higher risk of inadequate BMVA (*p* = 0.023) than primiparous women. SVA and BMVA were positively associated with DVA, especially for lactating women not using VA supplements (β = 0.174, 95%CI = 0.025, 0.324, *p* = 0.022; β = 0.501, 95%CI = 0.208, 0.795, *p* = 0.001). There was no association between SVA and BMVA (β = 0.165, 95%CI = −0.037, 0.366, *p* = 0.109). In conclusion, VA nutritional status among Chinese urban lactating women needs more attention, especially for those who are obese, overweight, or higher parity. Increased DVA may contribute to increased BMVA.

## 1. Introduction

Vitamin A (VA) plays an essential role in the regulation of gene expression and cell differentiation (retinoic acid), as well as in the functionality of vision, immune system and the health of the mucous epithelium [1]. VA deficiency continues to be one of the most significant public health problems in developing countries, and lactating women and infants are high-risk groups due to their increased demand for VA [2]. Newborn hepatic reserves of VA are low at birth to avoid possible teratogenic effects, so VA in breast milk is crucial, especially for exclusively breastfed infants [3,4]. Retinol concentrations in breast milk are used as a biomarker of VA deficiency in both lactating women and infants [5,6]. Previous studies have shown that the retinol concentration of breast milk differs widely among different countries, and the mean retinol concentration in the mature milk of women in developing countries is lower than the levels seen among women in developed countries [7]. VA deficiency is still a health issue that afflicts infants in China, the largest developing country, possibly due to lower retinol concentrations in breast milk [8].

In addition to breast milk vitamin A (BMVA), dietary vitamin A (DVA) and serum vitamin A (SVA) are crucial indicators reflecting a mother’s VA nutritional status, and the associations between the three remain a topic of discussion. One study reported poor correlations among DAV, SVA, and BMVA [9]. Other studies have reported that DVA intake is an essential factor of BMVA, and VA-fortified oil positively impacts BMVA [10,11]. However, some studies have inconsistent results [12]. Another study found that BMVA changes throughout lactation and reflects lower DVA intake, but SVA remains constant, imposing deficiency risks on breastfed infants [11]. Such findings challenge lactating women and infants’ nutritional education, and more evidence is needed.

In this study, we recruited lactating women from ten cities in China and collected their dietary information, blood samples, and breast milk samples. We aimed to investigate DVA intake, SVA concentrations, and BMVA concentrations of urban lactating Chinese women and explore the associated factors.

## 2. Materials and Methods

### 2.1. Study Site and Sampling

This study was based on a cross-sectional survey in urban China from 2019 to 2020. According to their economic and geographical characteristics, ten cities (Beijing, Guangzhou, Chengdu, Suzhou, Ningbo, Nanchang, Shenyang, Lanzhou, Hohhot, and Xuchang) were selected. In each city, one hospital-based maternal and child health care center was selected. Lactating women were conveniently recruited at the puerperal visit. The target sample size was at least 30 per city. The inclusion criteria were women at 30–90 days postpartum, aged between 20–45 years, with full-term singleton delivery, no smoking or alcohol abuse, and exclusively breastfeeding. The exclusion criteria were women with mastitis or any infectious diseases, severe mental diseases, and cardiovascular or metabolic diseases. We also excluded participants with missing values and extreme values. Data from 326 women were included in the current analyses.

### 2.2. Data Collection

Data were collected from lactating women by trained interviewers using an interviewer-administered questionnaire. The interviewers were trained and qualified in a standardized manner. Double-entry data and logical verification were conducted.

#### 2.2.1. Dietary Data

Dietary information was collected by a one-time 24-h dietary recall. Lactating women were asked to recall all food, beverages, and condiments consumed individually on the day prior to the survey. Standard bowls, plates, and spoons, as well as a picture booklet of the common foods consumed in China, were used to help participants to assess quantities [13]. DVA (μg RAE/day) (RAE stands for retinol activity equivalents) was calculated based on the Chinese Food Composition Table [14]. The recommended nutrient intake (RNI) for lactating women is 1300 μg RAE/day [15]. The following seven food groups were used to explore the dietary source of VA: (1) cereals and potatoes, (2) vegetables, (3) fruits, (4) meat (pork, beef, lamb, chicken, fish, seafood, animal organ, etc.) and eggs, (5) dairy products, (6) soybeans and nuts, and (7) others.

#### 2.2.2. Blood and Breast Milk Sample Collection and Laboratory Analysis

All participants were advised to follow their regular dietary habits before blood and breast milk collection. On the day of the survey, participants were asked to fast overnight for at least eight hours, feed their children, and empty their breasts before 7 a.m. A 5 mL blood sample was drawn, and serum was extracted. Then, milk samples were collected between 9 a.m. and 11 a.m. The breast on one side was cleaned using clean water and wiped with sterile gauze. The trained interviewers or mother herself squeezed the milk of a single breast into a sterile bottle. The bottle was then inverted 5–6 times to mix the foremilk, mid-milk, and hindmilk by hand, and a 35 mL sample was retained. Blood and breast milk samples were protected from direct light, stored at −20 °C at the local hospital, and transferred at −80 °C to the laboratory in Beijing. SVA (retinol) and BMVA (retinol) concentrations were assessed with high-performance liquid chromatography (Waters ACQUITY UPLC I-Class) by the internal standard method (retinyl acetate as the internal standard substance [16]). For SVA, we transferred 200 μL of the sample into a 2 mL EP tube, added 100 μL of the internal standard solution, and mixed it by shaking. We added 1200 μL of n-hexane extract, shook and mixed it thoroughly and then centrifuged the mixture. We transferred 800 μL of the supernatant into a 2 mL EP tube or a 96-well plate, dried it with nitrogen, reconstituted it in 100 μL of acetonitrile, shook and mixed it thoroughly, centrifuged it, then removed the supernatant for LC-MS/MS detection. The chromatographic column was ACQUITY UPLC^®^ BEH C18, 1.7 μm 2.1 × 50 mm, the column temperature was 40 °C, the mobile phase was 0.1% formic acid aqueous solution and 0.1% formic acid methanol (containing 2 mmol/L ammonium acetate), and the flow rate was 0.4 mL/min. For BMVA, we transferred 200 μL of the sample into a 1.5 mL EP tube, added 20 μL of internal standard solution, then added 20 μL of 10% ascorbic acid water and 200 μL of 1% BHT ethanol, vortexed it for 10 s to mix, added 50 μL of 5 M sodium hydroxide solution, and then added 50 μL of 5 M sodium hydroxide solution again. We vortexed and mixed the solution for 10 s, then place it into a water bath at 80 °C for 30 min saponification, and at 15 min, we took out the EP tube and mixed and shook it once to make the saponification uniform and complete. After the saponification was completed, we removed the solution and cooled it down, added 800 μL of n-hexane extract, vortexed and mixed it, centrifuged it, transferred 700 μL of supernatant into a 1.5 mL EP tube or a 96-well plate, dried it with nitrogen, and added 100 μL of 0.1% formic acid acetonitrile solution. The formic acid acetonitrile solution was redissolved, mixed and shaken, and detected on the machine. The chromatographic column was Accucore AQ 2.6 μm 50 × 2.1 mm, the column temperature was 40 °C, the mobile phase was 0.1% formic acid aqueous solution and 0.1% formic acid methanol (containing 2 mmoL/L ammonium acetate), and the flow rate was 0.4 mL/min. The laboratory analysis was conducted by a single qualified laboratory (Beijing Health Clinic Laboratory, China). VA concentrations below 0.7 and 1.05 μmol/L in serum and breast milk, respectively, were indicative of low VA nutritional status [12,17].

#### 2.2.3. Anthropometric Measurements and Assessments

For lactating women, weight (kg) and height (cm) were measured on the survey day, and pre-pregnancy weight (kg) and pre-delivery weight (kg) were self-reported. Body mass index (BMI) was calculated, and BMI < 18.5, 18.5–23.9, 24–27.9, and ≥28 kg/m^2^ were considered underweight, normal weight, overweight, and obese, respectively, according to the Chinese BMI standard [18]. Gestational weight gain (GWG) (kg) was calculated as the difference in pre-delivery weight and pre-pregnancy weight. According to the Chinese standard, GWGs for different pre-pregnancy BMIs is classified into three groups: insufficient, appropriate, and excessive [19].

#### 2.2.4. Other Covariates

Essential characteristics of the lactating women were collected, including sociodemographic characteristics (age, education, and family monthly per capita income), delivery information (parity and delivery mode), and infant information (sex and age). The metabolic equivalent of energy (MET) hours per week was calculated based on the short version of the International Physical Activity Questionnaire (IPAQ) [20]. Information on nutrient supplements during lactation was also collected. VA supplements were defined as those that contained VA and/or carotenoids. Since most of the lactating women could not recall the exact brand and dosage of VA supplements, this variable was dichotomous (yes/no), and the amounts of VA and/or carotenoids in them was not known.

### 2.3. Statistics

SPSS 26.0 and GraphPad Prism 8.0 were used for the analyses. Normality for continuous data was tested before the analyses. Because DVA, SVA, and BMVA did not fit the normal distribution, they were presented as medians (25th, 75th) and analyzed with Mann–Whitney U or Kruskal–Wallis H tests. Categorical variables were presented as percentages and analyzed with Chi-square tests. Spearman correlation tests were used to assess the correlations of DVA, SVA, and BMVA with postpartum days. Multiple regression analyses were used to assess the association among DVA, SVA, and BMVA. Model 0 was not adjusted. Model 1 was adjusted for possible influential variables (with *p* < 0.1 in the univariate analyses). Additionally, stratified analyses by VA supplements were performed. Differences were considered statistically significant at *p* < 0.05 (two-sided).

## 3. Results

### 3.1. DVA, SVA, and BMVA Status

The medians (25th, 75th) of DVA, SVA, and BMVA were 372.36 (209.12, 619.78) μg RAE/day, 1.99 (1.71, 2.35) μmol/L, and 1.67 (1.13, 2.15) μmol/L, respectively.

**Figure 1:**
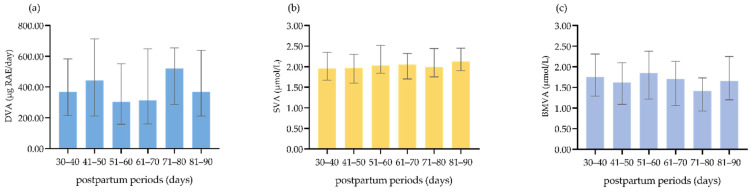
(**a**) DVA at different postpartum periods; (**b**) SVA at different postpartum periods; (**c**) BMVA at different postpartum periods.

DVA, SVA and BMVA status at different postpartum periods are presented in Figure 1. Spearman correlation analyses showed and DVA, SVA, and BMVA were not different at different postpartum periods (for DVA, *r* = −0.011, *p* = 0.847; for SVA *r* = 0.097, *p* = 0.079; for BMVA *r* = −0.072, *p* = 0.192). Kruskal–Wallis H tests showed the same results (for DVA, *p* = 0.984; for SVA, *p* = 0.210; for BMVA, *p* = 0.189).

### 3.2. Dietary Sources of VA

**Figure 2:**
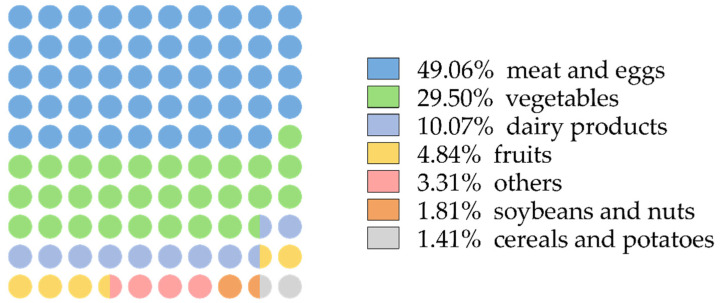
Dietary sources of VA of lactating women.

Nearly half of the VA was obtained from meat and eggs, followed by vegetables and dairy products (Figure 2).

### 3.3. DVA, SVA, and BMVA among Lactating Women with Different Characteristics

BMI categories distributed DVA, SVA, and BMVA differently (Table 1). Overweight or obese lactating women had lower DVA intake and BMVA concentrations but higher SVA concentrations. Nearly one-tenth of lactating women reported using VA supplements, but SVA and BMVA were not associated with it (*p* = 0.090; *p* = 0.602).

Only 3.1% of the lactating women met the RNI. All the lactating women had adequate SVA concentrations (≥0.7 μmol/L). Around one-fifth of lactating women (20.6%) had a BMVA less than 1.05 μmol/L. As shown in Table 2, lactating women with higher parity and BMI had lower proportions of achieving the recommended concentrations of BMVA.

### 3.4. Associations among DVA, SVA, and BMVA

**Figure 3:**
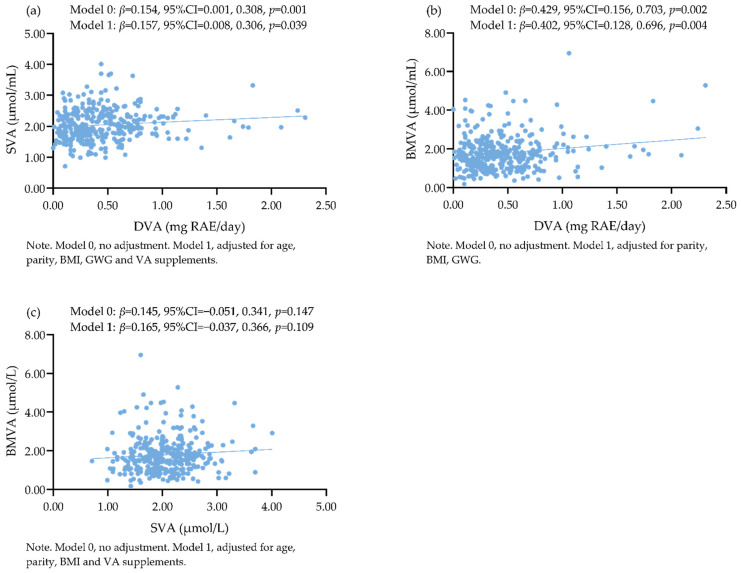
(**a**) Associations between DVA and SVA; (**b**) Associations between DVA and BMVA; (**c**) Associations between SVA and BMVA.

As shown in Figure 3, higher SVA and BMVA concentrations were associated with higher DVA intake after adjusting for possible influential factors. However, no association was observed between SVA and BMVA.

The results of the stratified analyses are presented in Table 3 and Table 4. For women not using VA supplements, SVA and BMVA were positively associated with DVA.

## 4. Discussion

Adequate nutritional VA status is crucial for the health and development of mother–infant pairs [21]. To the best of our knowledge, this is the first study to collect dietary information, blood samples, and breast milk samples of lactating women simultaneously in urban China. We found that only a tiny proportion of lactating women met the RNI of VA, and meat and eggs, vegetables, and dairy products were the essential sources of VA. Although the SVA levels of all of the lactating women were within normal limits, nearly one-fifth of them did not have the recommended levels of BMVA. One interesting result was that, compared to underweight or normal weight women, overweight or obese lactating women had lower DVA intake and BMVA concentrations but higher SVA concentrations. Moreover, multiparous women had a higher risk of inadequate BMVA (below 1.05 μmol/L) than primiparous women. Regarding associations among DVA, SVA, and BMVA, SVA and BMVA were positively associated with DVA, especially for lactating women not using VA supplements. There was no association between SVA and BMVA.

### 4.1. VA Intake of Urban Lactating Chinese Women

The human organism cannot synthesize VA, so the cause of VA deficiency has been ascribed mainly to inadequate VA intake [22]. Animal products are VA’s richest natural dietary source, and some fruits and leafy vegetables can provide VA as provitamin A carotenoids [23,24]. Consistent with previous studies [25,26], we also found that the DVA intake of urban lactating Chinese women needs to be increased. In China, lactating women are usually asked to follow traditional dietary habits with a high intake of meat and eggs and a low intake of fresh fruit and vegetables for 30 days after childbirth [27]. They are also usually asked to consume kinds of soups, such as millet soup and chicken soup, which are thought to be easy to digest and help with milk production. After 30 days postpartum, the degree of special care and attention given to the diet of lactating women is reduced, and the dietary quality changes, with less animal products (eggs, meat, dairy products) [28]. As a result, their DVA intake is also reduced. We also collected data on the use of VA supplements. However, because most of the lactating women could not recall the exact brand and dosage of their VA supplements, we could only determine a proportion of VA supplements users (12%). Since VA supplements should be used with caution [1,29], a VA-rich diet seems to be a more recommended and sustainable way to improve VA nutritional status.

### 4.2. SVA and BMVA of Urban Lactating Chinese Women

SVA and BMVA are considered reliable markers of VA nutritional status on a population basis. The current study found that all lactating women showed SVA levels above the cut-off value for inadequacy risk. Still, nearly one-fifth of them appeared to be at an increased risk of VA deficiency from the perspective of BMVA. That is, lactating women with normal VA blood indicators may have low levels of VA in their breast milk. This undoubtedly exposes their infants to the risk of VA deficiency in respect to building up liver stores. This result was consistent with another study [11].

### 4.3. Related Factors of SVA and BMVA of Urban Lactating Chinese Women

One interesting finding of the current study was the inconsistent relationship between bodyweight and SVA and BMVA. One study reported that maternal BMI was not associated with serum or breast milk retinol concentrations [30]. Another study reported that retinol concentration was higher in the mature milk of women with normal pregestational weights [31]. The serum retinol level of obese children was reported to be higher than that of non-obese children, which may be related to dietary structure [32]. The current study found overweight or obese lactating women had lower BMVA concentrations but higher SVA concentrations than underweight or normal weight women. Therefore, we inferred that overweight or obese lactating women may be at increased risk of VA deficiency, although they had standard serological markers. It should be mentioned that overweight and especially obese people often underreport their food intake [33]. Additional research is needed on how the body weight of lactating women influences breast milk components.

One study reported that colostrum retinol was higher in multiparous mothers, possibly because previous lactation enables high mobilization of retinol reserves and their high rates of transfer to the mammary gland [34]. However, we found that multiparous women had a higher risk of inadequate BMVA (below 1.05 μmol/L). Repeated reproductive cycles deplete maternal stores of nutrients [35]. Although the results of the two studies are inconsistent, two studies [34,35] point to the importance of adequate hepatic VA reserves. Notably, in the current study, when treated as a continuous variable, the BMVA concentrations did not show a statistically significant difference with parity. Therefore, further in-depth studies are required.

We did not expect to find that VA supplement users would not show high levels of SVA and BMVA. The associations between supplements and VA nutritional status remain inconclusive [36,37,38,39]. In the current study, due to incomplete data reporting on VA supplements, we could not determine the contribution of these supplements, and variables in dichotomous categories might limit the true association.

### 4.4. Associations among DVA, SVA, and BMVA of Urban Lactating Chinese Women

The current study found positive associations of higher SVA and BMVA with DVA, consistent with many previous studies [40,41]. Maternal dietary intake is essential because mothers need to compensate for the losses of pregnancy and childbirth, promote physical recovery, and produce milk to nurse their infants. Low dietary intake means that hepatic retinol tends to be used. Sufficient DVA intake is necessary for VA expression in breast milk at adequate levels [42]. Food-based interventions through fortification, biofortification, or dietary diversification are considered sustainable approaches to improving VA nutritional status [43]. Health education should be strengthened to make dietary structure during lactation more reasonable and reduce the risk of VA deficiency in mother–infant dyads.

Consistent with some previous studies [17,44], we also did not find an association between SVA and BMVA before or after stratified analyses by VA supplements. BMVA is derived from maternal VA stores and immediate dietary intake, which is transferred directly to the mammary gland by chylomicrons; therefore, BMVA could vary substantially while SVA remains unchanged [4]. The concentration of retinol in breast milk decreases throughout lactation and remains relatively constant after falling from its highest levels in colostrum [45]. In this process, SVA stays stable to maintain normal physiological functions.

### 4.5. Limitations

The current study had some limitations. First, because the study’s design was cross-sectional, it was not easy to make causal inferences, and there were confounding factors that could not be controlled. Second, the sampling method was not randomized, so a selection bias may exist. Third, DVA was calculated based on a one-time 24-h dietary recall. Although interviews of lactating women by trained interviewers may help obtain more accurate dietary information [46], recall bias may exist. Furthermore, a one-time 24-h dietary recall method may not reflect long-term DVA intake status, and diet misreporting may occur. A three-day 24-h dietary record is recommended in future studies. Finally, we only assessed retinol concentrations, and other biological markers, including retinol-binding protein and carotenoids, may help better understand the VA nutritional status of lactating women.

## 5. Conclusions

In our studied populations, only a tiny proportion of lactating women met the RNI of VA, and nearly one-fifth of them had a BMVA level that was lower than recommended. Lactating women who are overweight, obese, and with higher parity may have a worse VA nutritional status. SVA and BMVA were positively associated with DVA, especially for lactating women not using VA supplements.

## Figures and Tables

**Table 1:** DVA [μg RAE/day, median (25th, 75th)], SVA [μmol/L, median (25th, 75th)] and BMVA [μmol/L, median (25th, 75th)] among lactating women with different characteristics.

Characteristics	n (%)	DVA	*p* ^a^	SVA	*p* ^a^	BMVA	*p* ^a^
Age (years)							
≤30	177 (54.3)	373.69 (208.89, 614.95)	0.781	1.94 (1.66, 2.32)	0.090	1.62 (1.07, 2.09)	0.109
>30	149 (45.7)	366.73 (212.45, 629.67)		2.04 (1.76, 2.40)		1.68 (1.23, 2.36)	
College or university							
No	74 (22.7)	350.63 (188.89, 597.49)	0.538	2.00 (1.75, 2.40)	0.405	1.68 (1.31, 2.27)	0.471
Yes	252 (77.3)	381.75 (210.50, 629.96)		1.99 (1.70, 2.34)		1.67 (1.08, 2.13)	
Family monthly per capita income (Chinese yuan)							
<5000	129 (39.6)	363.10 (198.61, 644.87)	0.955	2.01 (1.77, 2.35)	0.736	1.54 (1.08, 2.06)	0.288
5000~9999	137 (42.0)	391.56 (219.68, 603.55)		1.98 (1.70, 2.35)		1.72 (1.14, 2.25)	
≥10,000	60 (18.4)	365.11 (217.56, 603.99)		1.95 (1.69, 2.35)		1.68 (1.16, 2.35)	
Parity							
1	227 (69.6)	398.40 (213.20, 606.15)	0.964	1.98 (1.70, 2.32)	0.068	1.72 (1.18, 2.13)	0.177
≥2	99 (30.4)	351.08 (196.90, 650.04)		2.05 (1.76, 2.44)		1.53 (0.97, 2.20)	
Delivery mode							
Cesarean delivery	127 (39.0)	391.56 (175.60, 630.35)	0.872	1.99 (1.69, 2.35)	0.452	1.60 (1.06, 2.15)	0.613
Vaginal delivery	199 (61.0)	364.32 (222.96, 611.58)		1.99 (1.73, 2.35)		1.71 (1.25, 2.20)	
BMI							
Underweight or normal weight	206 (63.2)	409.39 (248.07, 644.65)	0.022	1.97 (1.70, 2.32)	0.030	1.73 (1.29, 2.27)	0.003
Overweight or obese	120 (36.8)	334.92 (163.73, 586.56)		2.10 (1.77, 2.47)		1.51 (1.03, 1.89)	
GWG (kg)							
Insufficient or appropriate	169 (52.2)	437.19 (244.42, 649.19)	0.072	1.99 (1.73, 2.35)	0.738	1.70 (1.16, 2.21)	0.362
Excessive	155 (47.8)	336.35 (189.80, 600.09)		1.99 (1.70, 2.35)		1.58 (1.08, 2.13)	
Physical activities							
Low	141 (43.3)	353.02 (207.41, 582.37)	0.515	2.02 (1.76, 2.38)	0.334	1.70 (1.07, 2.17)	0.868
Medium	92 (28.2)	409.39 (218.99, 649.59)		1.95 (1.67, 2.31)		1.60 (1.09, 2.11)	
High	93 (28.5)	411.49 (193.40, 635.16)		1.99 (1.68, 2.36)		1.69 (1.24, 2.18)	
VA supplements							
No	287 (88.0)	391.56 (209.41, 620.18)	0.272	1.98 (1.70, 2.35)	0.090	1.66 (1.13, 2.13)	0.602
Yes	39 (12.0)	311.05 (196.90, 619.65)		2.13 (1.82, 2.60)		1.77 (1.08, 2.36)	
Infant sex							
Boy	165 (50.6)	364.32 (181.70, 600.66)	0.336	1.98 (1.74, 2.33)	0.411	1.72 (1.28, 2.13)	0.187
Girl	161 (49.4)	398.40 (222.23, 631.16)		2.01 (1.69, 2.39)		1.54 (1.05, 2.23)	
Infant age (days)							
30~60	226 (69.3)	373.40 (207.88, 610.58)	0.984	1.98 (1.67, 2.35)	0.210	1.68 (1.16, 2.21)	0.189
60~90	100 (30.7)	365.11 (215.64, 647.15)		2.03 (1.77, 2.37)		1.57 (1.07, 2.04)	

Note. ^a^: Mann–Whitney U or Kruskal–Wallis H tests.

**Table 2:** Stratification of lactating women according to the recommended concentration of BMVA.

Characteristics	BMVA		*p* ^a^
	<1.05 μmol/L, *n* (%)	≥1.05 μmol/L, *n* (%)	
n	67	259	
Age (years)			
≤30	41 (23.2)	136 (76.8)	0.203
>30	26 (17.4)	123 (82.6)	
College or university			
No	13 (17.6)	61 (82.4)	0.470
Yes	54 (21.4)	198 (78.6)	
Family monthly per capita income (Chinese yuan)			
<5000	28 (21.7)	101 (78.3)	0.367
5000~9999	30 (21.9)	107 (78.1)	
≥10,000	9 (15.0)	51 (85.0)	
Parity			
1	39 (17.2)	188 (82.8)	0.023
≥2	28 (28.3)	71 (71.7)	
Delivery mode			
Cesarean delivery	28 (22.0)	99 (78.0)	0.594
Vaginal delivery	39 (19.6)	160 (80.4)	
BMI			
Underweight or normal weight	35 (17.0)	171 (83.0)	0.037
Overweight or obese	32 (26.7)	88 (73.3)	
GWG (kg)			
Insufficient or appropriate	33 (19.5)	136 (80.5)	0.694
Excessive	33 (21.3)	122 (78.7)	
Physical activities			
Low	33 (23.4)	108 (76.6)	0.182
Medium	19 (20.7)	73 (79.3)	
High	15 (16.1)	78 (83.9)	
VA supplements			
No	59 (20.6)	228 (79.4)	0.995
Yes	8 (20.5)	31 (79.5)	
Infant sex			
Boy	29 (17.6)	136 (82.4)	0.178
Girl	38 (23.6)	123 (76.4)	
Infant age (days)			
30~60	46 (20.4)	180 (79.6)	0.894
60~90	21 (21.0)	79 (79.0)	

Note. ^a^: Chi-square tests.

**Table 3:** Stratified analyses regarding the association of DVA with SVA.

	β	SE	*p*	95%CI
With VA supplements				
DVA	0.034	0.341	0.921	(−0.660, 0.728)
Without VA supplements				
DVA	0.174	0.076	0.022	(0.025, 0.324)

Note. Adjusted for age, parity, BMI, and GWG.

**Table 4:** Stratified analyses regarding the association of DVA and SVA with BMVA.

	β	SE	*p*	95%CI
With VA supplements				
DVA ^a^	−0.432	0.398	0.285	(−1.241, 0.376)
SVA ^b^	0.026	0.036	0.469	(−0.046, 0.098)
Without VA supplements				
DVA ^a^	0.501	0.149	0.001	(0.208, 0.795)
SVA ^b^	0.136	0.116	0.244	(−0.093, 0.364)

Note. ^a^: adjusted for parity, BMI, and GWG; ^b^: adjusted for age, parity, and BMI.

## Data Availability

The raw data supporting the conclusions of this article will be made available by the authors, without undue reservation.
